# Can polygenic risk scores help explain disease prevalence differences around the world? A worldwide investigation

**DOI:** 10.1186/s12863-023-01168-9

**Published:** 2023-11-20

**Authors:** Pritesh R. Jain, Myson Burch, Melanie Martinez, Pablo Mir, Jakub P. Fichna, Cezary Zekanowski, Renata Rizzo, Zeynep Tümer, Csaba Barta, Evangelia Yannaki, John Stamatoyannopoulos, Petros Drineas, Peristera Paschou

**Affiliations:** 1 Department of Biological Sciences, Purdue University, West Lafayette, IN USA; 2 Department of Computer Sciences, Purdue University, West Lafayette, IN USA; 3 Unidad de Trastornos del Movimiento, Instituto de Biomedicina de Sevilla (IBiS). Hospital Universitario Virgen del Rocío/CSIC/Universidad de Sevilla, Seville, Spain; 4 Centro de Investigación Biomédica en Red Sobre Enfermedades Neurodegenerativas (CIBERNED), Madrid, Spain; 5 Department of Neurogenetics and Functional Genomics, Mossakowski Medical Research Institute, Polish Academy of Sciences, Warsaw, Poland; 6 Child and Adolescent Neurology and Psychiatry, Department of Clinical and Experimental Medicine, University of Catania, Catania, Italy; 7 Department of Clinical Genetics, Kennedy Center, Copenhagen University Hospital, Rigshospitalet, Copenhagen, Denmark; 8 Department of Clinical Medicine, Faculty of Health and Medical Sciences, University of Copenhagen, Copenhagen, Denmark; 9 Department of Molecular Biology, Institute of Biochemistry and Molecular Biology, Semmelweis University, Budapest, Hungary; 10 Hematology Department- Hematopoietic Cell Transplantation Unit, Gene and Cell Therapy Center, George Papanikolaou Hospital, Thessaloniki, Greece; 11 Department of Medicine, University of Washington, Seattle, WA USA; 12 Altius Institute for Biomedical Sciences, Seattle, WA USA; 13 Department of Genome Sciences, University of Washington, Seattle, WA USA; 14 Department of Medicine, Division of Oncology, University of Washington, Seattle, WA USA

**Keywords:** GWAS, PRS, Polygenic risk score, Ancestry, Disease prevalence

## Abstract

Complex disorders are caused by a combination of genetic, environmental and lifestyle factors, and their prevalence can vary greatly across different populations. The extent to which genetic risk, as identified by Genome Wide Association Study (GWAS), correlates to disease prevalence in different populations has not been investigated systematically. Here, we studied 14 different complex disorders and explored whether polygenic risk scores (PRS) based on current GWAS correlate to disease prevalence within Europe and around the world. A clear variation in GWAS-based genetic risk was observed based on ancestry and we identified populations that have a higher genetic liability for developing certain disorders. We found that for four out of the 14 studied disorders, PRS significantly correlates to disease prevalence within Europe. We also found significant correlations between worldwide disease prevalence and PRS for eight of the studied disorders with Multiple Sclerosis genetic risk having the highest correlation to disease prevalence. Based on current GWAS results, the across population differences in genetic risk for certain disorders can potentially be used to understand differences in disease prevalence and identify populations with the highest genetic liability. The study highlights both the limitations of PRS based on current GWAS but also the fact that in some cases, PRS may already have high predictive power. This could be due to the genetic architecture of specific disorders or increased GWAS power in some cases.

**Supplementary Information:**

The online version contains supplementary material available at 10.1186/s12863-023-01168-9.

## Introduction

Complex disorders are caused by the interaction of genetic, environmental and lifestyle factors. Most disorders that are frequent in human populations are complex [1] and their prevalence varies greatly around the world [2]. Understanding the basis of this prevalence difference can help disentangle the interaction among different factors causing complex disorders and identify groups of individuals who may be at a greater risk of developing certain disorders. This could become the basis of the implementation of early intervention strategies for populations at higher risk, with significant benefits for public health. To date, no systematic analyses have been performed to explore the possible correlation between genetic risk for complex disorders and their prevalence across populations.

The genetic component underlying complex disorders is not easy to quantify. It is highly polygenic in nature, possibly involving hundreds of genetic variants, each with a very small effect on disease liability and occurrence [3]. To measure the genetic risk of developing a specific disorder, it is possible to combine the effects of genomewide individual variants deriving a polygenic risk score (PRS) to quantify the genetic liability of a disorder and compare the risk of developing complex disorders across various populations [4]. The PRS of an individual for a specific disorder is estimated by the sum of multiple risk alleles, each weighted by the effect size of a specific allele [5], which is typically obtained from Genome-Wide Association Studies (GWAS). With the availability of large-scale datasets, thousands of GWAS have been performed for various traits and conditions, thus providing a large database of effect sizes that can be used to estimate PRS for a variety of complex disorders [6].

PRS have become an increasingly powerful tool to help identify individuals at higher risk of developing complex disorders and could also help explain the proportion of genetic variance that seems to be missing when focusing only on genome-wide significant hits [7, 8], as is common in GWAS. However, to date, PRS-based research has been hampered by the lack of GWAS summary statistics from diverse populations. It was recently highlighted that about 70% of GWAS studies since 2008 have used samples solely from European populations [9]. Previous studies have shown that the predictive power of PRS based on European GWAS is comparatively lower when applied to non-European populations and this decline increases as the target population diverges from the genetic structure observed in European populations [10]. The loss in prediction accuracy could be due to linkage disequilibrium (LD) structure and allele frequency differences between populations, which in turn could lead to differences in the effect size estimates from the GWAS based on one population compared to another [10–12].

Systematic studies attempting to evaluate the degree to which PRS can predict disease prevalence in different populations have not been performed to date in Europeans or non-Europeans. If such correlation of PRS to the epidemiology exists, it would significantly boost confidence in the validity of GWAS results and the potential for their use as a tool in the design of public health studies. Furthermore, in the case of non-Europeans, given the lack of large-scale GWAS data, the above-mentioned observations, and known differences in LD structure around the world one would expect poor transferability of findings. It is thus important to explore the relevance (if any) of European GWAS findings to non-Europeans.

Here, we embark on a systematic exploration of the genetic architecture of 14 complex disorders, by using large scale GWAS studies to estimate average genetic risk within Europe as well as around the world. We find that PRS significantly correlates to disease prevalence difference for four disorders within Europe. Extending our study to global populations, we find that PRS also correlates significantly with worldwide prevalence for eight disorders. We show that this correlation might be explained by the genetic architecture of the specific disorders and the potential conservation of genetic regions that have been implicated in disease susceptibility via GWAS. Our study highlights the validity of GWAS results and the important contribution of genetic background in shaping disease prevalence around the world.

## Results

### Complex disorders – prevalence and heritability

14 complex disorders grouped into five general categories (cardiovascular, neurological, autoimmune, metabolic, and psychiatric) were chosen for this analysis. The choice of disorders was based on both availability of large-scale GWAS data and disease prevalence, focusing on some of the most frequent diseases around the world with a large impact on public health. The genetic architecture for most of these disorders has been studied with large-scale GWAS analysis and for six of the 14 disorders, (Type 2 diabetes (T2D), chronic kidney disease (CKD), major depression (MDD), schizophrenia (SCZ), rheumatoid arthritis (RA) and asthma (AST)), we were able to obtain trans-ethnic GWAS results. For the rest of the disorders, GWAS studies based on individuals of European ancestry were used [13–26]. Table 1 shows the brief overview of the disorders being studied, including the global prevalence (obtained from the global burden of diseases dataset [2]) and the SNP heritability estimate of the diseases estimated from the GWAS summary statistics.

**Table 1:** List of studied disorders and sample size of the respective GWAS studies

Category	Disorder	GWAS Pop. (Eur/Trans)	World Prevalence	SNP Heritability	Study References
**Cardiovascular**	Coronary Artery Disease (CAD)	Eur	2.65	0.099	[14]
**Neurological**	Alzheimer’s Disease (AD)	Eur	0.69	0.0145	[13]
	Parkinson’s Disease (PD)	Eur	0.11	0.0113	[15]
**Metabolic**	Type 2 Diabetes (T2D)	Trans	5.89	0.0286	[16]
	Obesity (OBY)	Eur	12.23	0.1547	[17]
	Chronic Kidney Disease (CKD)	Trans	9.37	0.0246	[18]
**Autoimmune**	Asthma (AST)	Trans	3.53	0.075	[19]
	Type 1 Diabetes (T1D)	Eur	0.30	NA	[23]
	Rheumatoid Arthritis (RA)	Trans	0.25	0.143	[22]
	Crohn’s Disease (CRD)	Eur	0.07	0.86	[20]
	Multiple Sclerosis (MS)	Eur	0.02	0.0492	[21]
**Psychiatric**	Bipolar Disorder (BPD)	Eur	0.53	0.3	[24]
	Schizophrenia (SCZ)	Trans	0.32	0.157	[25]
	Major Depressive Disorder (MDD)	Trans	2.49	0.0214	[26]

**Fig. 1:**
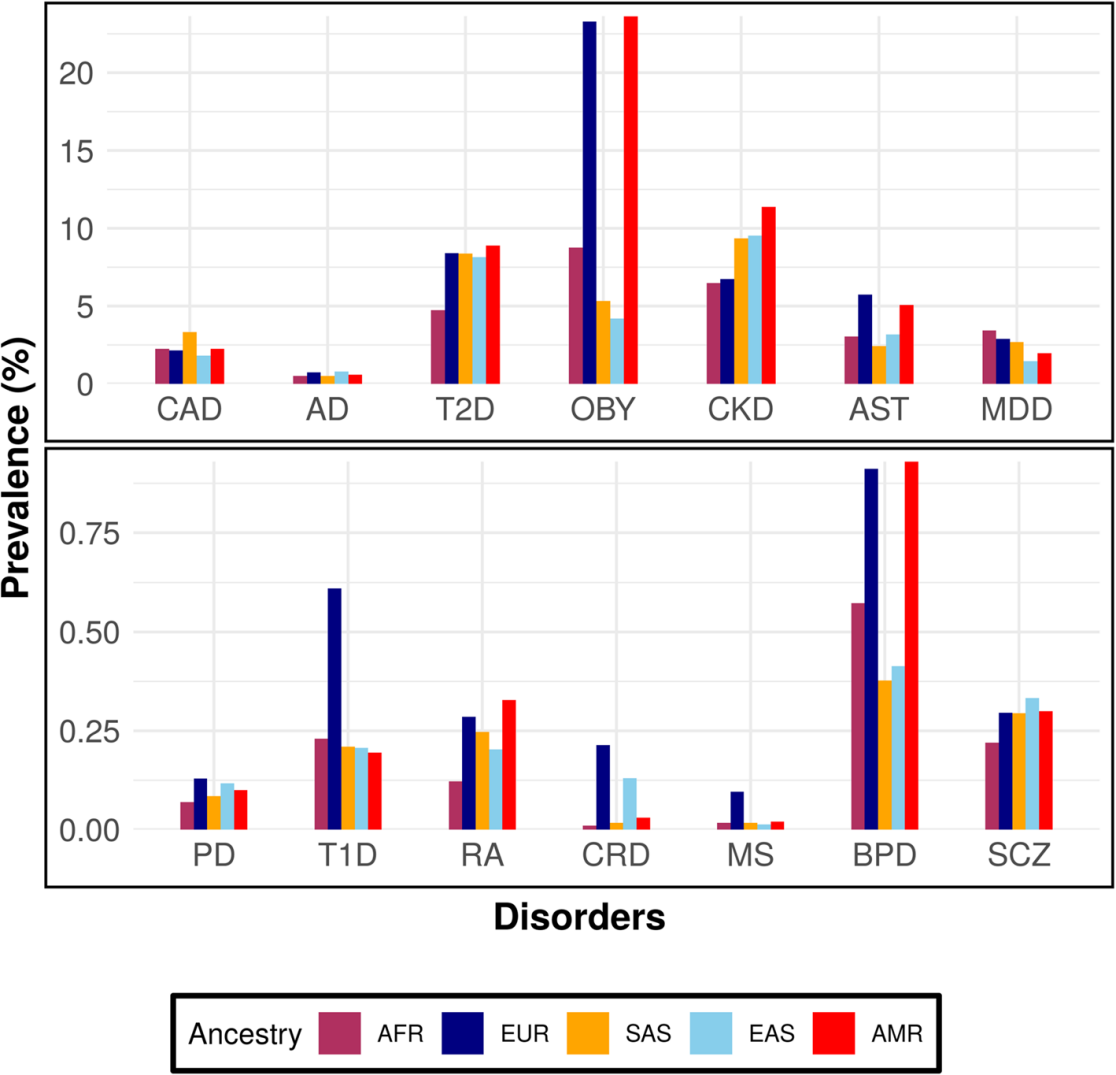
Bar plot showing the mean prevalence of 14 disorders across five ancestral groups. The x-axis indicates the ancestral group starting with Africans (AFR) and followed by Europeans (EUR), South Asians (SAS), East Asians (EAS), and admixed Americans (AMR). The y-axis is the mean prevalence (%) of each group calculated based on the different nationalities in each group

Among the 14 diseases studied, Metabolic disorders such as obesity, CKD and T2D have the highest global prevalence with values of 12.23%, 9.37% and 5.89% respectively. While comparing the prevalence estimates across the 5 super populations, Europeans had the highest prevalence of disorders like Parkinson disease (PD), AST, type 1 Diabetes (T1D) and crohn’s disease (CRD), while the prevalence of metabolic disorders was highest in admixed American populations (Fig. 1). We then use prevalence estimates for each (Additional file 1) to understand its correlation with genetic risk of the disorders across European and world populations.

### PRS of complex disorders in European populations

**Fig. 2:**
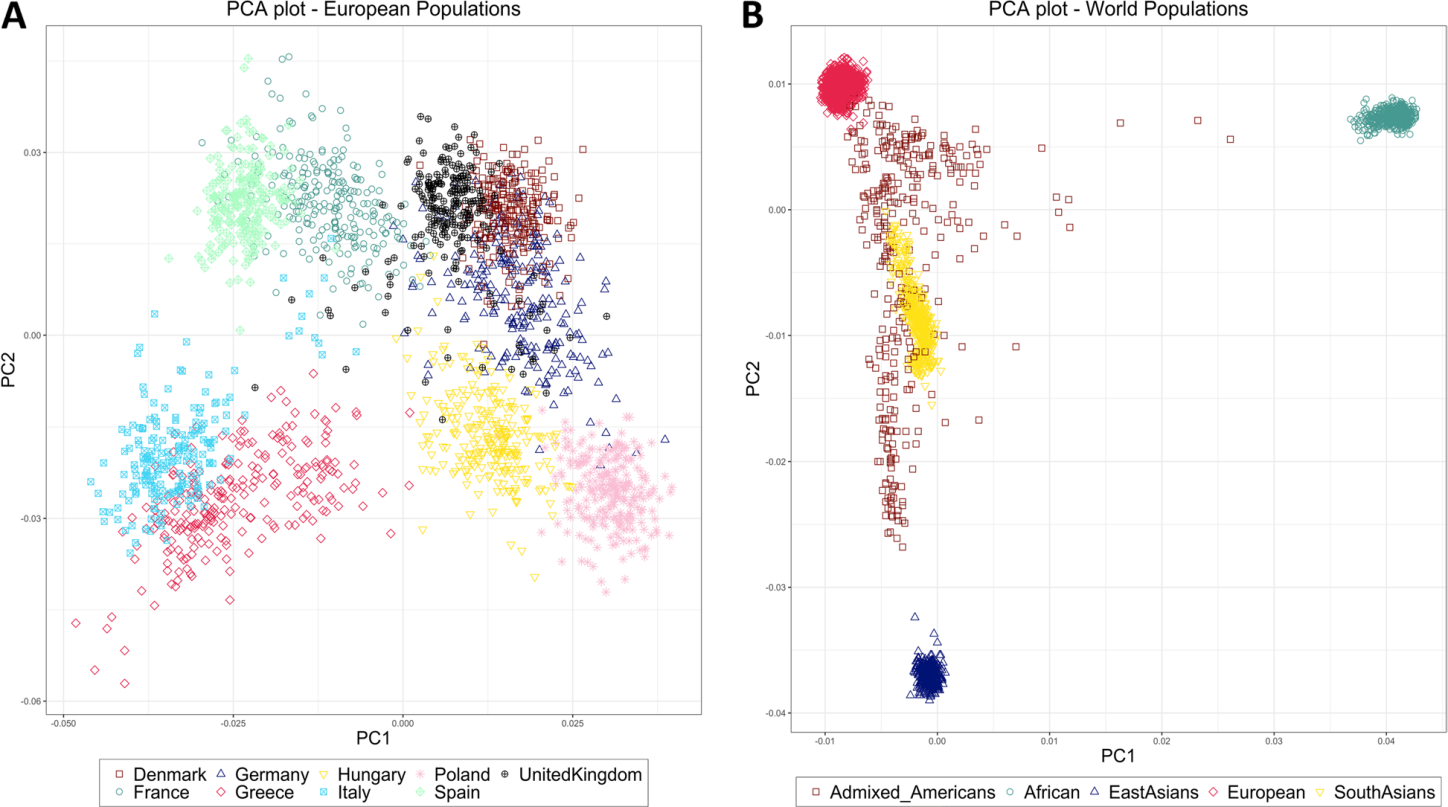
PCA plot of European and worldwide populations. The left panel (**A**) shows distribution of 2,109 European samples based on the top two principal components (PCs), colored and shaped based on their country of origin. The right panel (**B**) shows the distribution of 3,953 global samples based on the top two PCs, colored and shaped based on their region/ethnicity

We began by exploring PRS across a dataset of nine different European populations (2,109 individuals) obtained from previously published studies [27–30]. We computed the unweighted PRS (Supplementary Table 1) using plink2 score function [31] for the 14 different complex disorders (Table 1). The number of SNPs used for the PRS analysis are shown in Supplementary Table 2. The average scores for the 14 disorders across nine European populations are shown in Additional file 2. Principal component analysis (PCA) showed that the analyzed samples clustered based on geography (Fig. 2A). We calculated the correlation between the average PRS scores of each disorder and the average estimates of the top 2 principal components (PCs) of each of the 9 European populations. Although there was high correlation, the estimates were not significant for all disorders indicating that PRS does not always correlate with ancestry (Supplementary Table 3).

**Fig. 3:**
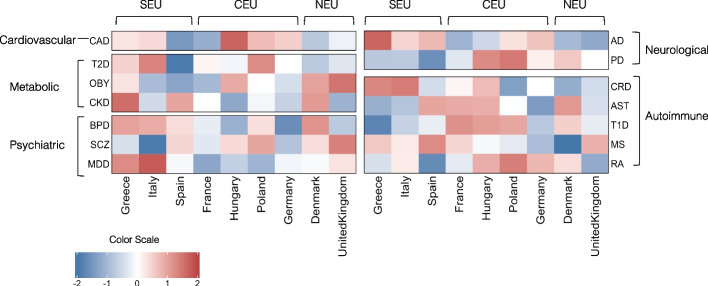
Heatmap of average PRS Scores (*p*-value < 5 × 10^–8^) of 14 disorders across European populations. The disorders are grouped based on the disease domain and the populations are arranged based on their geographical location, going from southern to northern countries. Shades of cells indicate the standardized average genetic risk of each disorder for each population. A higher risk is shown by red, and a lower risk is indicated by blue [SEU – South Europeans, CEU – Central Europeans, NEU – North Europeans]

Figure 3 shows the overall results at the threshold of genome wide significance (*p*-value < 5 × 10^–8^). Individuals from southern European countries (Greece and Italy) had a lower genetic risk of developing autoimmune disorders such as Asthma (AST) and Type 1 Diabetes (T1D) and higher genetic risk of developing Alzheimer’s Disease (AD), bipolar disorder and Major Depression (MDD), compared to central and northern European populations. The highest PRS for coronary artery disease (CAD) was observed in populations from central European countries like Hungary and Poland. These populations also showed a two-fold higher genetic risk for Parkinson disease (PD) and Rheumatoid Arthritis (RA), compared to other Europeans in this analysis. By contrast, we found that individuals from northern European countries like Denmark and the United Kingdom (UK) have lower genetic risk for neurological disorders and higher risk for disorders such as Obesity (OBESITY) and Schizophrenia (SCZ). The overall genetic risk of psychiatric disorders is lower in Central European populations.

To understand if genetic risk associated with disease prevalence, we calculated the correlation between the PRS and disease prevalence measures and, to test for statistical significance, we calculated an empirical *p*-value for association of significance based on random SNP sets as explained in methods; see Table 2A for our results. We observed significant correlation between prevalence and PRS for four disorders: CAD (*R*^2^ = 0.77, *p *= 0.004), T1D (*R*^2^ = 0.38, *p* = 0.03), obesity (*R*^2^ = 0.51, *p* = 0.016), and MDD (*R*^2^ = 0.50, *p* = 0.017).

**Table 2:** Pearson’s correlations coefficients between average genetic risk for complex disorders (calculated via PRS) and the actual prevalence of the respective disorders in (A) nine European populations and (B) 24 world populations. The values represent the correlation coefficient and *p*-value based on 1000 permutations (shown in parentheses). The (*) indicates an empirical *p*-value < 0.05, based on 100 random sets

Disorder	(A) Correlation between PRS and prevalence (Europeans only)		(B) Correlation between PRS and prevalence (World Populations)	
	**R2**	***p*-value**	**R2**	***p*-value**
CAD	**0.78**	0.014*	0.07	0.756
AD	0.21	0.591	-0.03	0.896
PD	0.32	0.52	**0.56**	0.004*
T2D	0.17	0.668	**0.63**	0.001*
OBY	**0.51**	0.016*	**0.68**	0.001*
CKD	-0.04	0.910	-0.31	0.136
CRD	-0.04	0.922	**0.58**	0.003*
AST	0.38	0.313	**0.51**	0.011*
T1D	**0.54**	0.013*	-0.04	0.867
MS	-0.22	0.564	**0.69**	0.001*
RA	-0.20	0.601	0.21	0.315
BPD	-0.30	0.441	-0.38	0.067
SCZ	-0.32	0.406	**0.64**	0.001*
MDD	**0.50**	0.017*	**0.65**	0.001*

### Extending current GWAS studies to global populations

Having found that PRS correlates to disease prevalence differences within Europe for CAD,T1D, MDD and obesity, we proceeded to investigate the extent of such correlations outside Europe. If such correlations are identified in some cases, our hypothesis is that this could signify that for some disorders, genomic regions that are less diverse around the world have been implicated. We expanded our analysis to global populations (European samples combined with 1000 genomes phase 3 data [32]). The overall data set included a total of 3,953 individuals from 24 different populations in five regions of the world (Supplementary Table 1). The PCA plot of the global data again showed that the populations are very tightly clustered based on their regions of origin, except for the AMR samples which are distributed along a cline (Fig. 2B).

**Fig. 4:**
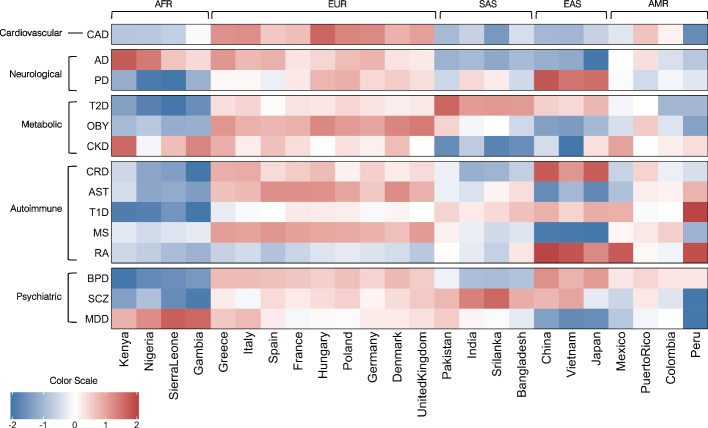
Heatmap of average PRS scores (*p*-value < 5 × 10^–8^) of 14 disorders across worldwide populations. The disorders are grouped based on the disease domain and the populations are arranged based on their geographical location and ancestry, starting with Africans (AFR) and followed by Europeans (EUR), South Asians (SAS), East Asians (EAS), and Admixed Americans (AMR). Shades of cells indicate the standardized average genetic risk of each disorder for each population. A higher risk is shown by red, and lower risk is indicated by blue

We calculated GWAS PRS scores for the global population samples (Additional file 3) using available data from trans-ethnic GWAS whenever available (for six disorders ie) and European ancestry GWAS for the rest (Table 1). In Fig. 4, we compared the average PRS calculated at a threshold of *p*-value < 5 × 10^–8^ for 24 populations from five regions of the world. Genetic risk for the different disorders was observed to follow a pattern that is reminiscent of geography. Indeed, populations originating from the same region tend to have a rather uniform genetic risk score, as compared to risk scores between populations from different regions. This was also validated with strong correlations observed between the average genetic risk score of each country and the mean PC1 and PC2 estimates of each population (Supplementary Table 4).

We observed that the mean PRS for AD, MDD and Chronic Kidney Disease (CKD) was highest in African populations. These populations also had a lower risk for most autoimmune disorders and other metabolic disorders such as obesity and Type 2 Diabetes (T2D). Asian populations including East Asians and South Asians had a higher genetic risk for T2D and a lower risk for obesity compared to other populations. Additionally, East Asians had the highest PRS scores for Crohn’s Disease (CRD) and Rheumatoid Arthritis (RA). European populations had the highest genetic risk scores for CAD and obesity and were at a moderate genetic risk for most autoimmune and psychiatric disorders compared to other world populations. The AMR populations had a high genetic risk estimates for disorders like bipolar disorder (BPD) and T1D.

### Correlation between PRS and prevalence of complex disorders around the world

The mean prevalence of the disorders across the five ancestral populations that we studied is shown in Fig. 1, while the prevalence of each disorder in each country is shown in Additional file 1 and the results of the correlation analysis are shown in Table 2B. Out of the 14 disorders, we found significant correlation between the disease prevalence for eight disorders with the respective PRS at a *p*-value threshold of 5 × 10^–8^. The strongest correlation was observed for MS (*R*^2^ = 0.69, *p* = 0.001). Other significant correlations were observed for autoimmune disorders including CRD (*R*^2^ = 0.58, *p* = 0.003), AST (*R*^2^ = 0.51, *p* = 0.013). We also observed significant correlation between the Average PRS and prevalence for metabolic disorders like obesity (*R*^2^ = 0.698 *p* = 0.001) and T2D (*R*^2^ = 0.63, *p* = 0.001) as well as psychiatric disorders like SCZ (*R*^2^ = 0.64, *p* = 0.001) and MDD (*R*^2^ = 0.65, *p* = 0.001).

### Genetic architecture of disease associated regions used for PRS analysis

The significant association between worldwide disease prevalence and PRS could be tied to the specific genetic architecture of these disorders as well as a strong genetic involvement in defining disease prevalence around the world. We hypothesize that this could be partially explained from biologically relevant signals identified by GWAS that are more conserved (reduced difference in frequency and LD structure) across world-wide populations compared to random SNPs**.** To test this hypothesis, we explored the worldwide structure and allele frequency differences of genomic regions that were used in our PRS analysis for all the disorders. First, we calculated r^2^ [33] for all pairs of variants within 100 kb of the PRS SNPs and we performed pairwise comparisons between Europeans and individuals from other geographic regions. Second, we calculated the mean F_ST_ of the PRS SNPs, again performing pairwise comparisons between European populations and other populations [34]. The empirical *p*-value was calculated using a statistical test based on random SNP sets (see Methods section for details).

#### LD – r2 analysis

**Fig. 5:**
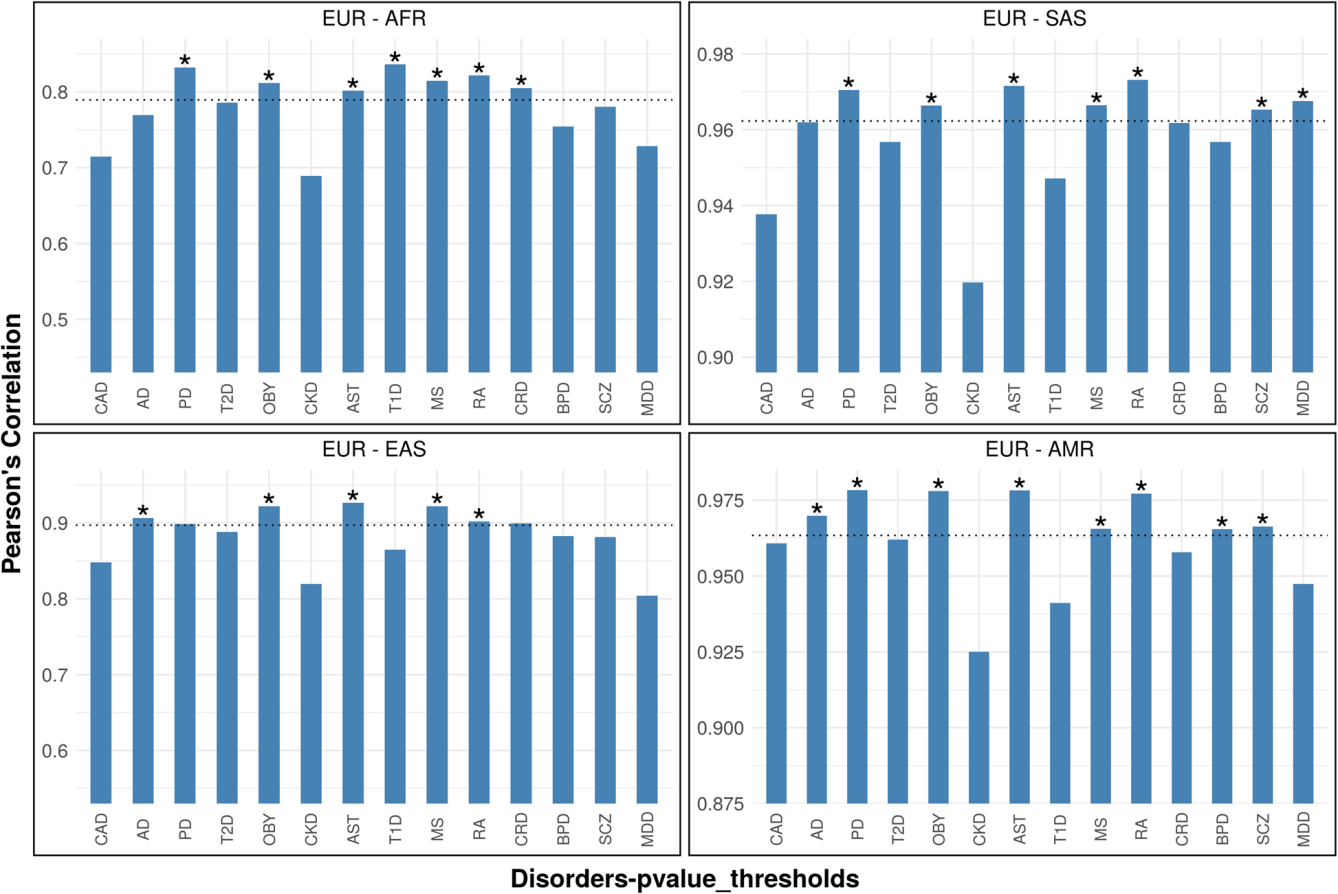
Bar plot showing Pearson's correlation coefficients between four pairs of populations. The x-axis indicates the disorders, and the y-axis shows the correlation coefficient between each pair of populations. The dotted line shows the mean correlation value of the distribution based on 100 randomly chosen SNP sets. (*) indicates empirical *p*-values below 0.05

Results of our LD—r^2^ analysis showed that for multiple studied disorders, the genetic regions used in PRS calculations show similar LD structure around the world compared to randomly selected regions (empirical *p*-value < 0.05), (see Fig. 5 and Supplementary Table 5). For instance, the regions around the genome-wide significant SNPs used in the computation of obesity, AST, MS, and RA PRS, revealed similar LD patterns across all populations, indicating that the associated loci have similar genetic structure across world-wide populations. We also observed conserved LD structure between African and European individuals for regions used in PRS computations for all autoimmune disorders and also for PD and T2D. The LD structure for regions used for PRS in South Asians (SAS) was significantly correlated with European structure for seven out of the fourteen disorders which included five disorders that had significant correlation between genetic risk and prevalence (namely obesity, asthma, MS, MDD and SCZ). Europeans and Asians were the most differentiated with only five disorders showing significantly similar LD patterns, whereas the comparison of LD structure between Admixed Americans and Europeans for the studied genetic regions showed significant correlation for nine of the fourteen disorders which indicated that these populations were the least differentiated.

#### FST analysis

F_ST_ analysis also revealed low genetic differentiation around the world for many of the genetic regions used for PRS computations (see Supplementary Fig. 1 and Supplementary Table 6). For instance, the SNPs used for PRS calculations for AST and MDD had significantly lower F_ST_ between Europeans and other worldwide populations, compared to randomly selected SNPs (empirical *p*-value < 0.05). Similar to the results obtained from the LD analysis, autoimmune disorders had a significantly lower F_ST_ between Africans and Europeans. The results of F_ST_ comparisons between SAS/AMR populations and Europeans were concordant with the results of the LD analysis in both cases with disorders like obesity, CRD, MDD and RA having significantly lower F_ST_.

### Sensitivity analysis

To better understand how associations are affected as a function of the *p*-value threshold used for the PRS calculations, we performed similar analyses at relaxed *p*-value thresholds in order to include more SNPs in the risk score calculation. Results for PRS calculated at other *p*-value thresholds revealed an overall similar distribution of disease risk. At higher *p*-value thresholds, the differences between populations became more pronounced and stronger clustering was observed between countries in the same region (see Supplementary Fig. 2 and Additional file 2) The distribution of PRS calculated for all worldwide populations indicated that the genetic risk distribution for certain disorders changes at different thresholds (Supplementary Fig. 3). The average scores for all disorders in global populations at all thresholds are listed in Additional file 3.

We then estimated the correlation between PRS at different thresholds and the prevalence of the studied disorders among European populations as well as worldwide populations. The results among European populations showed significant associations for CAD at different thresholds, as well as additional significant associations for other disorders like AD, RA, and MS (see Supplementary Table 7). Similarly, among worldwide populations, we observed that the associations were conserved for all seven disorders. Additionally, we found significant associations for disorders like CAD and BPD, which were not observed at *p*-value < 5 × 10^–8^ (see Supplementary Table 8).

## Discussion

The prevalence of complex disorders across different populations is often quite varied. This may be attributed to a combination of differences in genetic factors, lifestyle, and environment. To the best of our knowledge, no previous study to date has systematically investigated the correlation between genetic background and disease prevalence differences in Europe and around the world. In this work, we first explored the genetic component of this variation using PRS to determine and compare the average genetic risk of 14 disorders in individuals belonging to different populations within Europe. For six of the studied disorders trans-ethnic GWAS results were available. For the rest, we use GWAS conducted in individuals of European ancestry. In most cases, we observed clear differences in the distribution of the average PRS estimates based on ancestry. Interestingly, within Europe, we found significant correlation between genetic risk and disease prevalence for four disorders. We then expanded the analysis to understand the differences across world populations. The results showed a great degree of variation in genetic risk with populations belonging to the same ancestry groups having similar risk estimates. For eight out of the 14 studied disorders, we demonstrated statistically significant correlations between the average PRS and disease prevalence. obesity and MDD had significant correlations between genetic risk and prevalence in both Europe and around the world. Our results indicate that the differences in genetic predisposition of a disorder across populations may potentially help explain differences in disease prevalence across populations.

Polygenic risk scores can potentially be used to identify populations with high genetic predisposition for various disorders. For instance, the highest number of individuals with T2D throughout the world is reported in Asia. Here, we showed that East Asians had increased genetic risk for T2D [35]. It is also interesting that in Asian populations we found the genetic risk for obesity to be quite low, which could explain the unique clinical presentation of diabetic phenotype in Asian populations with lower rates of obesity [36]. Europeans have the highest lifetime prevalence of autoimmune disorders such as MS, T1D and RA as seen from the Global Burden of disease data [2]. In concordance with this observation, we found a higher genetic risk of developing autoimmune conditions in European populations compared to people of other ancestries.

The lack of non-European GWAS for many of the studied disorders is a limitation of our analysis, since there could be a decrease in the prediction accuracy of PRS as previously described [9–12]. However, despite this limitation, for disorders such as OBESITY, CRD and MS we found that average PRS of various non-European populations calculated using GWAS based on Europeans can actually still capture differences in disease prevalence across these populations. We also observed a very high correlation between average PRS and principal components across populations, which provides additional validation that the difference in prevalence across populations could be explained by the difference in genetic risk due to ancestry. We also showed a low differentiation of LD structure and allele frequency for regions around the SNPs used in PRS calculations for multiple disorders, suggesting that GWAS may be identifying disease-causing loci that are conserved across populations and have reduced difference in allele frequency compared to random SNPs.

Using GWAS based on both trans-ethnic and European individuals, we were able to capture genetic risk differences and correlations with prevalence around the world for eight disorders, including Obesity, Multiple Sclerosis, Crohn’s Disease, Type 2 Diabetes, Parkinson’s Disease, Asthma, Schizophrenia and Major Depression. However, when we used European GWAS studies, we may have missed variants that might be significantly associated with disease in non-European populations and not seen in Europe [10, 37]. Another limitation of our analysis is that the awareness regarding various conditions, especially psychiatric disorders, may be low in developing countries and, as a result, prevalence data might be biased [38]. This could also explain why fewer significant associations were observed for psychiatric disorders in non-European populations. Finally, the method used for PRS calculation uses a simple approach of selecting SNPs and does not consider the differences in genetic architecture of the world population. This could potentially bias the correlation estimates of disorders with weaker GWAS summary results as not all the disease associated loci would be used for the PRS calculations.

Identification of populations that carry increased genetic susceptibility to disease could help inform clinical practice and public health strategies. If certain populations have a higher risk of a specific disorders, earlier intervention strategies could be implemented and potentially be shaped into a public health policy. Additionally, as PRS scores become more accurate in disease prediction, it is possible to use them at an individual level from a personalized medicine approach to identify the genetic susceptibility to develop various complex disorders [39]. It is therefore of great importance to consider the relevance and transferability of findings to populations that differ from the original GWAS discovery populations. Finally, our work can also be expanded to study and identify individuals and populations who could be at a higher risk for severe symptoms due to specific environmental factors operating at different world regions [40].

Here, we provided evidence to support the validity of GWAS and the identification of loci that are biologically relevant and thus more conserved across populations. This, together with the specific genetic architecture of each disorder, could explain the correlation of PRS to worldwide disease prevalence that we observed for eight of the studied disorders. As more and more GWAS studies based on trans-ancestral populations become available, future studies in this direction could use these and apply novel methods for PRS calculations that can better adjust for differences in ancestry in base and target datasets by either modeling the LD structure or including annotation and fine-mapping data [41, 42]. With large trans-ethnic sample sizes in GWAS studies it is expected that the prediction accuracy of PRS will improve greatly, and the method can then be expanded to understand the genetic risk of traits across populations with no prevalence data. Ultimately, combining genetic risk, lifestyle information, and environmental factors will help elucidate differences in disease prevalence around the world and inform the design of future public health strategies.

## Conclusion

This is the first attempt to systematically the degree to which PRS can predict disease prevalence in different populations from around the world. We estimated the genetic risk of 14 complex disorders across five different continental regions to explore whether genetics might help explain disease prevalence distribution around the world. We found that PRS of world populations can indeed capture differences in disease risk and could thus be used to identify populations with the highest genetic liability to develop various disorders. Significant correlations were observed between genetic risk and disease prevalence for eight disorders in different global populations. Intriguingly, the genetic loci around the disease-associated SNPs showed similar LD patterns and allele frequencies around the world. The results of these analyses highlight the validity of GWAS results and could help inform clinical and public health decisions in populations with a higher genetic risk of developing different complex disorders.

## Methods

### Data sets

We collected publicly available GWAS summary statistics for 14 complex disorders with no overlap with the target data [13–26]. The disorders can be grouped in five general categories (cardiovascular, neurological, autoimmune, metabolic, and psychiatric). The data was cleaned to remove any duplicate and mismatched SNPs. The target dataset for the analysis consisted of 3,953 samples from 24 different countries belonging to five different ancestral groups: Africans (504), Europeans (2109), South Asians (489), East Asians (504) and Admixed Americans (347). The European samples were collected from previous studies [27–30] and the samples from other populations were acquired from the publicly available 1000 genomes phase 3 data [32]. The detailed list of data sources is shown in Supplementary Table 2 and all appropriate informed consent, IRB approvals, and Data Use Agreements are in place for use of data as part of this study. The dataset was cleaned using Plink [31] to filter out variants with more than 2% missingness, minor allele frequency < 0.01, and Hardy–Weinberg Equilibrium < 1e-6. After QC, we included 3,953 samples and 1,618,220 imputed SNPs for PRS calculation. The prevalence data for 14 traits was collected from the Global Burden of Disease (GBD) database [2] and the prevalence information for obesity was collected from the WHO [43] (see Additional file 1). For conditions like AD, CRD, and CAD for which specific data was not available, we used the prevalence data from broad traits like dementia, IBD, and ischemic heart disease.

### Principal component analysis

We performed principal component analysis for both the European and global dataset to visualize the genetic architecture of the different populations. The EIGENSOFT software which implements the Eigenstrat smartPCA method was used to run the analysis [44]. The dataset was cleaned to remove the MHC and the chromosome 8 inversion region. We applied LD pruning within a 100 KB region threshold and r^2^ of 0.1 to select independent SNP. Overall, 88,899 SNPs were used to calculate the Principal Components (PCs).

### Polygenic risk scores estimation

PRS is generally calculated as the sum of the number of risk alleles weighted by the effect of the allele for the specific disorder. In this case however, since we intend to calculate the PRS for individuals across various populations, the effect sizes may not be transferable [9, 10, 12]. To reduce such bias, we calculate an unweighted polygenic risk score for every individual based on the direction of association of each SNP (obtained from GWAS summary statistics) and hence the scores become a function of allele frequency across populations [45]. Independent SNPs were selected for each disorder with a clumping threshold (r^2^) of 0.1 within a 250 kb distance and *p*-value threshold of 5e-08. We then repeated this at four other *p*-value thresholds for our sensitivity analysis (1e-05, 0.001, 0.05, 1). The number of SNPs used at each threshold for each of the disorders are shown in Supplementary Table 3. The Plink score function is used to estimate the PRS of each individual and then the average PRS scores for the 24 countries are calculated to visualize the mean distribution pattern of the genetic risk of various disorders and identify populations with higher genetic risk. We also used these scores to estimate the correlation between genetic risk and prevalence of a disorder.

### Correlations with prevalence and empirical *p*-value calculations

To determine if the average genetic risk of a disorder in a population is associated with the prevalence of disorder, we estimated Pearson’s correlation coefficients between the Average scores and the prevalence data. To calculate the empirical *p*-value and confirm the significance, we performed a statistical test using a PRS method with random SNP selection. We first picked 100 random SNP sets to compute PRS with the number of SNPs in each set equal to the number of SNPs crossing the PRS significance threshold. We then computed the correlation coefficients between each random SNP set and the prevalence of the target disorder. This gives us a distribution of observed correlation coefficients between PRS and disease prevalence. The distribution was then used to determine the empirical *p*-value of by identifying the number of SNP sets that had significant correlation higher than the PRS scores at the actual threshold.

### Linkage Disequilibrium (LD) analysis

To determine if the regions around SNPs used for PRS calculations are conserved across populations, we extracted all variants within a 100 KB region around the PRS SNPs and calculated LD r^2^ [33] for all pairs of SNPs within the region. This was done independently for each of the five ancestral populations and was repeated for all disorders separately. We then compared the r^2^ values of the various pairs of SNPs in Europeans to the values of the same pair in each of the other four populations to estimate the Pearson’s correlation for each disorder. To calculate an empirical *p*-value, we first constructed 100 SNP sets, with each set having 1,000 SNPs selected randomly to understand whether the GWAS SNPs were more conserved than randomly selected SNPs. For each set, we then repeated the analysis as described above and obtained a distribution of correlation estimates. We then used this distribution to determine if the correlations observed between Europeans and each of the other populations for different disorders are significantly higher (top 5^th^ percentile) compared to the correlation distribution obtained from the random SNP sets. The estimation of r^2^ was done using the Plink tool and the statistical analyses were performed in R.

### FST Analysis

We selected SNPs that were used for PRS calculations and then estimated the F_ST_ [34] for four different groups composed of Europeans and Africans, Europeans and South Asians, Europeans and East Asians, and Europeans and Admixed Americans. We calculated the F_ST_ of the selected SNPs in each group individually, with each ancestry used as a sub-population, and determined the mean F_ST_ of all SNPs in each pair. Analysis was repeated separately for all disorders. To calculate an empirical *p*-value for both analyses, we created 100 sets of 1000 randomly selected SNPs and repeated the F_ST_ calculations to get a distribution. We used this distribution to verify if the mean F_ST_ of the PRS SNPs in each population pair is significantly lower (bottom 5^th^ percentile) than the distribution of the random SNP sets. The F_ST_ calculation was done using the Plink tool and the statistical analyses were performed in R.

### Supplementary Information

**Additional file 1.** Age adjusted prevalence data of the 14 disorders in 24 global populations.

**Additional file 2.** Average PRS of each disorder in the nine European populations. Each sheet represents the score calculated using SNPs at different p-value thresholds.

**Additional file 3.** Average PRS of each disorder in the 24 worldwide populations. Each sheet represents the score calculated using SNPs at different p-value thresholds.

**Additional file 4:** **Supplementary Table 1.** Data sources and number of samples analyzed per population. **Supplementary Table 2.** Number of SNPs used for PRS calculation. The first column indicates the disorder, and each following column indicates number of SNPs used in the estimation at different p-value thresholds. **Supplementary Table 3.** Pearson’s correlation coefficients between average genetic risk between 14 complex disorders and the average location of 9 European populations in a PCA plot (PC1 and PC2 only). The value in each cell represents the correlation coefficient and the respective p-value estimate. **Supplementary Table 4.** Pearson’s correlation coefficients between average genetic risk between 14 complex disorders and the average location of 24 world populations in a PCA plot (PC1 and PC2 only). The value in each cell represents the correlation coefficient and the respective p-value estimate. **Supplementary Table 5.** Pearson's correlation coefficients of r^2^ estimates of SNP pairs in regions used for PRS estimation between 4 pairs of populations. Statistically significant results (empirical *p*-value <0.05) are indicated as bold. **Supplementary Table 6.** Mean FST estimates of PRS SNPs between European and other populations for the six disorders that demonstrated significant correlation between average PRS and population prevalence. Statistically significant results (empirical *p*-value < 0.05) are indicated as bold. **Supplementary Table 7.** Pearson’s correlation coefficients for average genetic risk between 18 complex disorders and their prevalence in European populations. The column headers indicate the *p*-value threshold for PRS calculation and the value in each cell shows the correlation coefficient (R^2^) and respective *p*-value (in parentheses). (*) indicates empirical *p*-value<0.05. **Supplementary Table 8.** Pearson’s correlation coefficient for average genetic risk between 18 complex disorders and their prevalence in 24 countries. The column headers indicate the *p*-value threshold for PRS calculations. The value in each cell represents the correlation coefficients and *p*-values based on 1,000 permutations (shown in parentheses). (*) indicates empirical *p*-value<0.05. **Supplementary Figure 1.** Bar plot showing the mean F_ST_ between four pairs of populations. The x-axis indicates the disorders, and the y-axis shows the mean F_ST_ for each pair of populations. The dotted line shows the mean F_ST_ value of a distribution formed using 100 random SNP sets. (*) indicates an empirical *p*-value below 0.05. **Supplementary Figure 2.** Heatmap of average PRS (*r*^2^ = 0.1; *p*-value<1) of 14 Disorders across European Populations. Populations are arranged based on geographical proximity; shades of cells indicate the standardized genetic risk of each disorder for each population. A higher risk is shown by red, and a lower risk is indicated by blue [SEU – South Europeans, CEU – Central Europeans, NEU – North Europeans]. **Supplementary Figure 3.** Heatmap of average PRS (*r*^2^ = 0.1; *p*-value<1) of 14 Disorders across Worldwide Populations. Populations are arranged based on geographical proximity; shades of cells indicate the standardized genetic risk of each disorder for each population. A higher risk is shown by red, and a lower risk is indicated by blue. [AFR – Africans, EUR – Europeans, SAS – South Asians, EAS– East Asians, AMR – Admixed Americans].

## Data Availability

The genomic data used in this analysis can be obtained upon reasonable request to the authors. The non-European data used for the analysis was obtained and downloaded from the 1000 genomes phase 3 data. (https://www.internationalgenome.org/data). Other Sources include WTCCC (EGAS00000000028), Popgen Study, TS-EROTRAIN study, and the Three city study; access to which can be obtained upon request to authors. The GWAS summary statistics were downloaded from the GWAS Catalog (https://www.ebi.ac.uk/gwas/) and the GWAS Atlas (https://atlas.ctglab.nl/) which contains summary statistics information for different traits and disorders.. The prevalence data was obtained from the global burden of diseases 2019 data resource (https://ghdx.healthdata.org/gbd-2019).

## Abbreviations

GWAS: Genome Wide Association studies
PRS: Polygenic Risk Scores
LD: Linkage disequilibrium
CAD: Coronary Artery Disease
AD: Alzheimer’s Disease
PD: Parkinson’s Disease
T2D: Type 2 Diabetes
CKD: Chronic Kidney Disease
OBY: Obesity
T1D: Type 1 Diabetes
AST: Asthma
RA: Rheumatoid Arthritis
CRD: Crohn’s Disease
MS: Multiple Sclerosis
BPD: Bipolar Disorder
SCZ: Schizophrenia
MDD: Major Depressive Disorder
EUR: European
AFR: African
EAS: East Asian
SAS: South Asian
AMR: Admixed American
