# Supplementary material for: Can polygenic risk scores help explain disease prevalence differences around the world? A worldwide investigation
Source: BMC Genom Data. 2023 Nov 20;24:70. doi: 10.1186/s12863-023-01168-9 (PMC10662565; doi:10.1186/s12863-023-01168-9)
Supplement: Supplementary file 4 — Additional file 4: Supplementary Table 1. Data sources and number of samples analyzed per population. Supplementary Table 2. Number of SNPs used for PRS calculation. The first column indicates the disorder, and each following column indicates number of SNPs used in the estimation at different p-value thresholds. Supplementary Table 3. Pearson’s correlation coefficients between average genetic risk between 14 complex disorders and the average location of 9 European populations in a PCA plot (PC1 and PC2 only). The value in each cell represents the correlation coefficient and the respective p-value estimate. Supplementary Table 4. Pearson’s correlation coefficients between average genetic risk between 14 complex disorders and the average location of 24 world populations in a PCA plot (PC1 and PC2 only). The value in each cell represents the correlation coefficient and the respective p-value estimate. Supplementary Table 5. Pearson's correlation coefficients of r2 estimates of SNP pairs in regions used for PRS estimation between 4 pairs of populations. Statistically significant results (empirical p-value <0.05) are indicated as bold. Supplementary Table 6. Mean FST estimates of PRS SNPs between European and other populations for the six disorders that demonstrated significant correlation between average PRS and population prevalence. Statistically significant results (empirical p-value < 0.05) are indicated as bold. Supplementary Table 7. Pearson’s correlation coefficients for average genetic risk between 18 complex disorders and their prevalence in European populations. The column headers indicate the p-value threshold for PRS calculation and the value in each cell shows the correlation coefficient (R2) and respective p-value (in parentheses). (*) indicates empirical p-value<0.05. Supplementary Table 8. Pearson’s correlation coefficient for average genetic risk between 18 complex disorders and their prevalence in 24 countries. The column headers indicate the p-value t [file 12863_2023_1168_MOESM4_ESM.pdf]

Supplement to "Can polygenic risk scores predict disease prevalence differences around the world? A worldwide investigation."

Jain et al.

List of Tables

1 Sources of population and sample sizes ……………………………………….. 2

2 Number of SNPs used for PRS calculation ……………………………………. 3

3 Correlation between Avg. PRS and Avg. PCs within Europe………………… 4

4 Correlation between Avg. PRS and Avg. PCs for all populations……………. 5

5 Correlations between r^2 estimates of EUR and other populations …………... 6

6 F_{ST} estimates between EUR and other populations …………………………... 7

7 Correlations between PRS and prevalence in European populations ……… 8

8 Correlations between PRS and prevalence in Global populations…………... 9

List of Figures

1 Mean F_{ST} between Europeans and other populations………………………… 10

2 PRS Distribution across European Populations (PRS p-value <1) ………….. 11

3 PRS Distribution across World Populations (PRS p-value <1) ……………… 12

References …………………………………………………………………………….. 13

Supplementary Table 1: Data sources and number of samples analyzed per population.

Super Population	Country	Sample Size	Data Source
Europeans	Greece	246	Paschou et al., PNAS (1)<br>Paschou et al., Annals of Neurology (2)<br>Stamatoyannopoulos et al., EJHG (3)
	Italy	192	
	Hungary	249	
	Poland	249	TS – EUROTRAIN Study (4)
	Spain	222	
	Denmark	244	
	Germany	246	Popgen Study (5)
	France	244	Three city study(6)
	United Kingdom	217	WTCCC(code : EGAS00000000028) (7)
Africans	Kenya	99	1000 Genomes project (8)
	Nigeria	207	
	Sierra Leone	85	
	Gambia	113	
South Asians	Pakistan	96	
	India	205	
	Srilanka	102	
	Bangladesh	86	
East Asians	China	301	
	Vietnam	99	
	Japan	104	
Admixed Americans	Mexico	64	
	Puerto Rico	104	
	Colombia	94	
	Peru	85	

Supplementary Table 2: Number of SNPs used for PRS calculation. The first column indicates the disorder, and each following column indicates number of SNPs used in the estimation at different p-value thresholds.

**Disorders**	**p < 5E-08**	**p < 5E-05**	**p < 0.001**	**p < 0.01**	**p < 0.05**	**p < 1**
CAD	33	150	519	2308	7850	63824
AD	23	80	394	2294	8173	63972
PD	17	61	368	2279	8146	64218
T2D	136	414	1271	4068	10574	64163
OBY	16	72	343	1649	5735	52337
CKD	17	85	552	2924	9480	64062
AST	146	327	1291	4230	11024	63905
T1D	26	68	146	357	802	6703
RA	33	76	388	1985	6834	60157
CRD	88	274	872	3460	10354	63725
MS	15	76	270	1255	4656	42901
BPD	3	75	792	3406	9869	61602
SCZ	182	571	2484	6538	14382	62841
MDD	1	21	450	2603	8701	61035

Supplementary table 3: Pearson's correlation coefficients between average genetic risk between 14 complex disorders and the average location of 9 European populations in a PCA plot (PC1 and PC2 only). The value in each cell represents the correlation coefficient and the respective p-value estimate.

**Disorder**	**R2 (PC1)**	**p value (PC1)**	**R2 (PC2)**	**p value (PC2)**
CAD	0.37	0.328	-0.77	0.015
AD	-0.40	0.287	-0.57	0.112
PD	0.88	0.002	-0.30	0.425
T2D	0.05	0.899	-0.75	0.020
OBY	0.54	0.137	-0.06	0.881
CKD	-0.35	0.351	0.06	0.888
CRD	-0.69	0.040	-0.46	0.212
AST	-0.24	0.526	0.28	0.467
T1D	0.51	0.157	0.23	0.553
MS	-0.61	0.083	-0.03	0.931
RA	0.61	0.081	-0.55	0.128
BPD	0.44	0.236	0.19	0.622
SCZ	0.58	0.104	0.27	0.479
MDD	-0.64	0.062	-0.32	0.401

Supplementary table 4: Pearson's correlation coefficients between average genetic risk between 14 complex disorders and the average location of 24 world populations in a PCA plot (PC1 and PC2 only). The value in each cell represents the correlation coefficient and the respective p-value estimate.

Disorder	R2 (PC1)	p value (PC1)	R2 (PC2)	p value (PC2)
CAD	-0.41	0.048	0.67	0.001
AD	0.34	0.103	0.84	0.001
PD	-0.73	0.001	-0.52	0.009
T2D	-0.77	0.001	-0.31	0.146
OBY	-0.59	0.002	0.66	0.001
CKD	0.29	0.166	0.51	0.011
CRD	-0.63	0.001	-0.34	0.109
AST	0.46	0.023	0.85	0.001
T1D	-0.79	0.001	-0.54	0.006
MS	-0.31	0.135	0.89	0.001
RA	-0.27	0.207	-0.85	0.001
BPD	0.83	0.001	0.24	0.253
SCZ	-0.64	0.001	-0.19	0.375
MDD	0.54	0.007	0.78	0.001

Supplementary table 5: Pearson's correlation coefficients of r^2 estimates of SNP pairs in regions used for PRS estimation between 4 pairs of populations. Statistically significant results (empirical p-value <0.05) are indicated as bold.

**Disorder**	**EUR – AFR**	**EUR – SAS**	**EUR – EAS**	**EUR – AMR**
**Mean (Random Set)**	0.789	0.962	0.897	0.963
CAD	0.715	0.938	0.848	0.961
AD	0.770	0.962	**0.907**	**0.970**
PD	**0.832**	**0.971**	0.899	**0.978**
T2D	0.786	0.957	0.888	0.962
OBY	**0.811**	**0.966**	**0.922**	**0.978**
CKD	0.689	0.920	0.819	0.925
CRD	**0.805**	0.962	0.900	0.958
AST	**0.801**	**0.972**	**0.927**	**0.978**
T1D	**0.836**	0.947	0.865	0.941
MS	**0.815**	**0.966**	**0.922**	**0.966**
RA	**0.821**	**0.973**	**0.902**	**0.977**
BPD	0.754	0.957	0.883	**0.965**
SCZ	0.780	**0.965**	0.882	**0.966**
MDD	0.729	**0.968**	0.804	0.947

Supplementary table 6: Mean F_{ST} estimates of PRS SNPs between European and other populations for the six disorders that demonstrated significant correlation between average PRS and population prevalence. Statistically significant results (empirical p-value < 0.05) are indicated as bold.

**Disorder**	**EUR – AFR**	**EUR – SAS**	**EUR – EAS**	**EUR – AMR**
**Mean (Random Set)**	0.108	0.033	0.085	0.024
CAD	0.128	0.056	0.096	0.045
AD	0.129	0.034	**0.061**	**0.015**
PD	0.121	0.056	0.079	**0.020**
T2D	**0.106**	0.041	0.102	0.031
OBY	0.112	**0.021**	**0.063**	**0.019**
CKD	**0.021**	0.060	0.207	0.052
CRD	0.108	**0.017**	0.089	**0.021**
AST	**0.052**	**0.017**	**0.066**	**0.015**
T1D	0.158	**0.026**	0.170	0.030
MS	0.123	0.045	0.156	0.040
RA	**0.070**	0.027	0.132	0.042
BPD	0.118	0.059	0.124	**0.011**
SCZ	0.146	0.041	0.105	0.028
MDD	**0.029**	**0.002**	**0.070**	**0.023**

Supplementary table 7: Pearson's correlation coefficients for average genetic risk between 18 complex disorders and their prevalence in European populations. The column headers indicate the p-value threshold for PRS calculation and the value in each cell shows the correlation coefficient (R2) and respective p-value (in parentheses). **(*)** indicates empirical p-value<0.05.

**Disorder**	**Pt<5x10^{-05}**		**Pt<0.001**		**Pt<0.05**		**Pt<1**	
	**R2**	**p-val**	**R2**	**p-val**	**R2**	**p-val**	**R2**	**p-val**
CAD	0.48	0.189	0.58	0.098	0.07	0.853	-0.08	0.835
AD	0.12	0.758	-0.30	0.427	-0.22	0.573	0.42	0.026*
PD	0.20	0.89	-0.34	0.369	0.23	0.546	0.11	0.769
T2D	0.10	0.783	-0.14	0.721	-0.13	0.733	0.12	0.766
OBY	0.37	0.032*	0.66	0.043*	0.04	0.917	-0.07	0.851
CKD	0.43	0.241	-0.11	0.782	-0.14	0.712	-0.30	0.438
CRD	0.17	0.656	0.16	0.673	0.13	0.735	-0.09	0.809
AST	-0.012	0.976	-0.37	0.334	-0.37	0.332	-0.21	0.592
T1D	0.78	0.012	-0.26	0.500	-0.54	0.129	-0.59	0.096
MS	0.06	0.863	0.09	0.814	0.30	0.028*	0.42	0.264
RA	-0.13	0.722	0.35	0.358	0.41	0.003*	0.47	0.200
BPD	0.49	0.177	0.19	0.633	-0.11	0.777	-0.12	0.761
SCZ	-0.11	0.766	0.40	0.289	0.75	0.021*	0.80	0.009
MDD	0.32	0.400	-0.41	0.271	-0.27	0.490	-0.03	0.929

Supplementary Table 8: Pearson's correlation coefficient for average genetic risk between 18 complex disorders and their prevalence in 24 countries. The column headers indicate the p-value threshold for PRS calculations. The value in each cell represents the correlation coefficients and p-values based on 1,000 permutations (shown in parentheses). **(*)** indicates empirical p-value<0.05.

Disorder	$Pt<1x10^{-05}$		Pt<0.001		Pt<0.05		Pt<1	
	R2	p-val	R2	p-val	R2	p-val	R2	p-val
CAD	-0.04	0.847	0.14	0.501	-0.04	0.861	-0.06	0.791
AD	0.13	0.548	-0.18	0.404	0.50	0.013	0.47	0.021
PD	0.44	0.032*	-0.36	0.082	-0.36	0.089	-0.61	0.002
T2D	0.58	0.003	-0.19	0.374	-0.40	0.054	-0.60	0.002
OBY	0.76	0.001*	0.28	0.203	-0.64	0.001	-0.66	0.001
CKD	-0.23	0.287	-0.30	0.149	-0.02	0.936	-0.09	0.667
CRD	0.62	0.001*	-0.03	0.903	-0.47	0.019	-0.35	0.091
AST	0.04	0.848	0.05	0.830	0.49	0.015*	0.49	0.015
T1D	-0.30	0.150	-0.50	0.012	-0.52	0.008	-0.46	0.024
MS	0.56	0.005*	-0.33	0.115	0.11	0.617	0.68	0.001
RA	0.20	0.353	0.03	0.898	-0.54	0.006	-0.46	0.023
BPD	-0.54	0.007*	-0.08	0.713	0.32	0.013	0.29	0.177
SCZ	0.59	0.002*	-0.50	0.012	-0.69	0.001	-0.74	0.000
MDD	0.35	0.095	0.48	0.016*	-0.12	0.573	-0.45	0.029

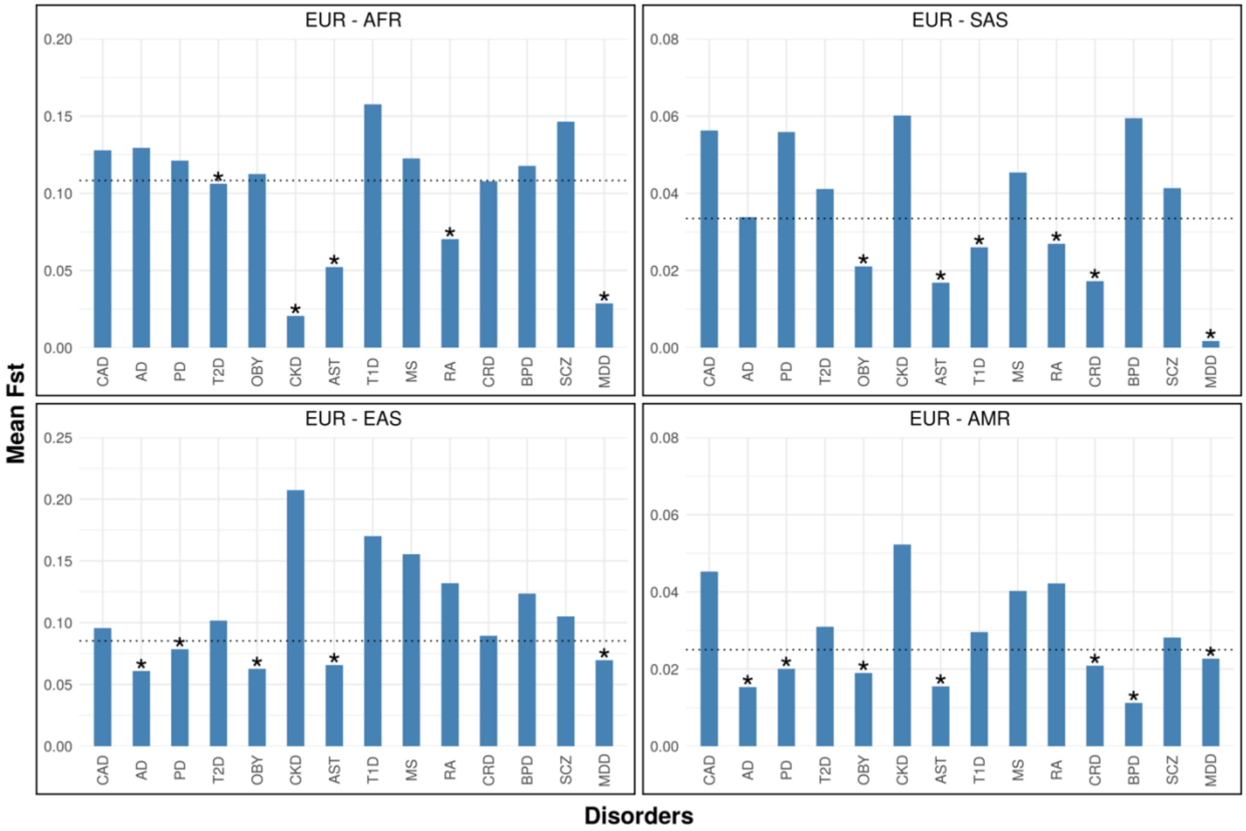

Supplementary Figure 1: Bar plot showing the mean F_{ST} between four pairs of populations. The x-axis indicates the disorders, and the y-axis shows the mean F_{ST} for each pair of populations. The dotted line shows the mean F_{ST} value of a distribution formed using 100 random SNP sets. (*) indicates an empirical p-value below 0.05.

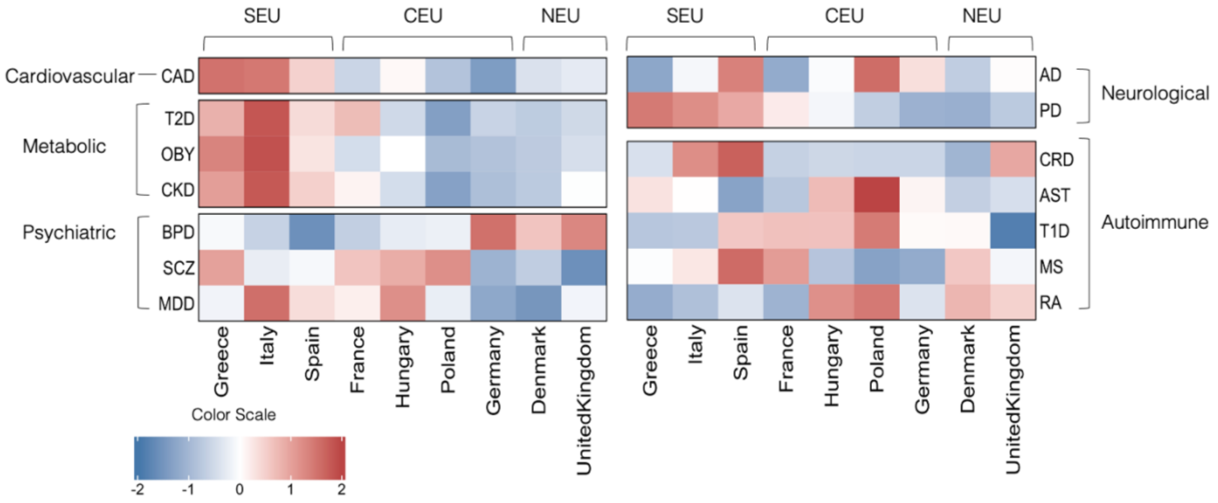

Supplementary Figure 2: Heatmap of average PRS (r^2 = 0.1; p-value<1) of 14 Disorders across European Populations. Populations are arranged based on geographical proximity; shades of cells indicate the standardized genetic risk of each disorder for each population. A higher risk is shown by red, and a lower risk is indicated by blue [SEU – South Europeans, CEU – Central Europeans, NEU – North Europeans].

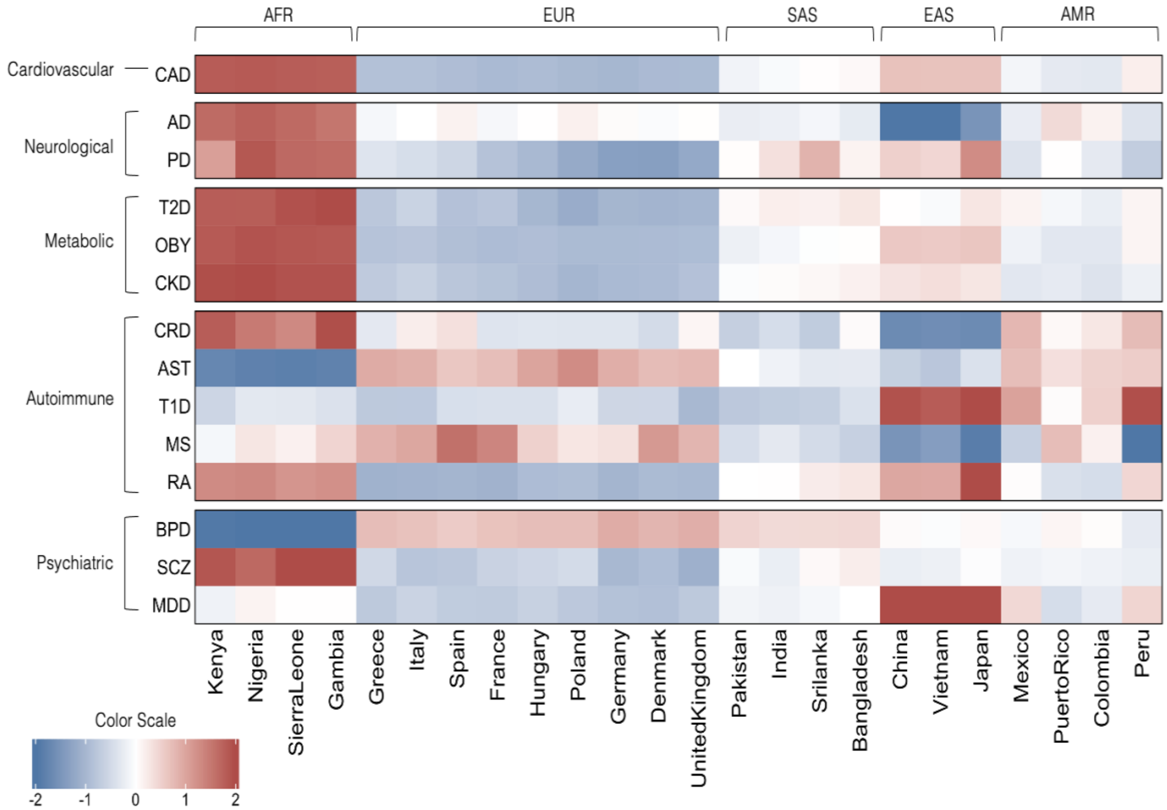

Supplementary Figure 3: Heatmap of average PRS (r^2 = 0.1; p-value<1) of 14 Disorders across Worldwide Populations. Populations are arranged based on geographical proximity; shades of cells indicate the standardized genetic risk of each disorder for each population. A higher risk is shown by red, and a lower risk is indicated by blue. [AFR – Africans, EUR – Europeans, SAS – South Asians, EAS – East Asians, AMR – Admixed Americans]

References

1. Paschou P, Drineas P, Yannaki E, Razou A, Kanaki K, Tsetsos F, et al. Maritime route of colonization of Europe. Proc Natl Acad Sci . 2014 Jun 24
2. Paschou P, Yu D, Gerber G, Evans P, Tsetsos F, Davis LK, et al. Genetic association signal near NTN4 in Tourette syndrome. Ann Neurol. 2014 Aug 1 [cited 2021 Nov 5];76(2):310–5
3. Stamatoyannopoulos G, Bose A, Teodosiadis A, Tsetsos F, Plantinga A, Psatha N, et al. Genetics of the peloponnesean populations and the theory of extinction of the medieval peloponnesean Greeks. Eur J Hum Genet 2017 255. 2017 Mar 8 [cited 2021 Nov 5];25(5):637–45.
4. Forde NJ, Kanaan AS, Widomska J, Padmanabhuni SS, Nespoli E, Alexander J, et al. TS-EUROTRAIN: A European-Wide Investigation and Training Network on the Etiology and Pathophysiology of Gilles de la Tourette Syndrome. Front Neurosci. 2016 Aug 23;0(AUG):384.
5. U N, M K. [PopGen. A population-based biobank with prospective follow-up of a control group]. Bundesgesundheitsblatt Gesundheitsforschung Gesundheitsschutz. 2012 Jun [cited 2021 Oct 11];55(6–7):831–5.
6. Antoniak M, Pugliatti M, Hubbard R, Britton J, Sotgiu S, Sadovnick AD, et al. Vascular Factors and Risk of Dementia: Design of the Three-City Study and Baseline Characteristics of the Study Population. Neuroepidemiology. 2003;22(6):316–25.
7. Burton PR, Clayton DG, Cardon LR, Craddock N, Deloukas P, Duncanson A, et al. Genome-wide association study of 14,000 cases of seven common diseases and 3,000 shared controls. Nature. 2007
8. Auton A, Abecasis GR, Altshuler DM, Durbin RM, Bentley DR, Chakravarti A, et al. A global reference for human genetic variation. Vol. 526, Nature. Nature Publishing Group; 2015
